# TransLiG: a de novo transcriptome assembler that uses line graph iteration

**DOI:** 10.1186/s13059-019-1690-7

**Published:** 2019-04-23

**Authors:** Juntao Liu, Ting Yu, Zengchao Mu, Guojun Li

**Affiliations:** School of Mathematics, Shandong University, Jinan, 250100 China

**Keywords:** RNA-seq data, Transcriptome assembly, Splicing graph, Line graph, Algorithm

## Abstract

We present TransLiG, a new de novo transcriptome assembler, which is able to integrate the sequence depth and pair-end information into the assembling procedure by phasing paths and iteratively constructing line graphs starting from splicing graphs. TransLiG is shown to be significantly superior to all the salient de novo assemblers in both accuracy and computing resources when tested on artificial and real RNA-seq data. TransLiG is freely available at https://sourceforge.net/projects/transcriptomeassembly/files/.

**Electronic supplementary material:**

The online version of this article (10.1186/s13059-019-1690-7) contains supplementary material, which is available to authorized users.

## Background

Alternative splicing is an important form of genetic regulation in eukaryotic genes, increasing the gene functional diversity as well as the risk of diseases [1–3]. As reported [4, 5], most of the eukaryotic genes including human genes undergo the process of alternative splicing, and so one gene could produce tens or even hundreds of splicing isoforms in different cellular conditions, causing different functions and potential diseases. Therefore, the identification of all the full-length transcripts under specific conditions plays a crucial role in many subsequent biological studies. However, we are still far from a complete landscape of human transcripts, and the situation is even much less clear for non-human eukaryotic species [6].

RNA-seq is a powerful technology that enables the identification of expressed genes as well as abundance measurements at the whole transcriptome level with unprecedented accuracy [7–10]. The RNA-seq protocol takes as input the sampled expressed transcripts and produces more than 200 million short reads for a run, and each sequencing read is generally of length 50–150 base pairs, posing great challenges to reconstruct the full-length transcripts from the RNA-seq reads. Firstly, different transcripts could have highly different expression abundances, which makes the constructed sequence graph (splicing graph, *de bruijn* graph, etc.) have quite uneven coverage. Secondly, different transcripts from the same gene can share exonic sequences due to alternative splicing, making the splicing graph even more complicated. Thirdly, a large amount of RNA-seq reads contain sequencing errors, making it more difficult to assemble those lowly expressed transcripts from the RNA-seq data. All of the above have made the transcriptome assembly problem highly challenging.

There have been a growing number of methods developed for solving the transcript assembly problem in recent years, and most of them could be categorized into two approaches: the reference-based (or genome-guided) and the de novo [11, 12]. The reference-based approaches such as Scallop [13], TransComb [14], StringTie [6], Cufflinks [15], and Scripture [16] usually first map the RNA-seq reads to a reference genome using alignment tools such as Hisat [17], Star [18], Tophat [19], SpliceMap [20], MapSplice [21], or GSNAP [22], and the reads from the same gene locus would fall into a cluster to form a splicing graph, and all the expressed transcripts could be assembled by traversing the graphs. The assembled transcripts by this strategy generally have higher accuracy compared to those by de novo strategy as it benefits from a reference genome, but it is seriously limited in practice because such a high-quality reference genome is currently unavailable for most species.

De novo assembly is a desired approach when the reference genome is unavailable, incomplete, highly fragmented, or substantially altered as in cancer tissues. There have been a number of de novo assemblers, such as BinPacker [23], Bridger [24], Trinity [12], IDBA-Tran [25], SOAPdenovo-trans [26], ABySS [27], and Oases [28]. This strategy usually directly constructs splicing graphs from RNA-seq reads based on their sequence overlaps, and then assembles transcripts by traversing the graphs using different algorithms. Assemblers like IDBA-Tran, SOAPdenovo-trans, ABySS, and Oases were developed based on the key techniques in genome assembly, and so in general, they do not work well in transcriptome assembly. Trinity opens the door to design a method specifically for handling the de novo assembly of transcriptome. It first extends the sequencing reads into long contigs by a k-mer extension strategy, then connects those contigs into a *de bruijn* graph, and finally infers all the expressed transcripts by traversing the *de bruijn* graph. As noticed in the Trinity paper, there are some limitations hindering its applications. The sequence depth information which would be useful in the assembling procedure was not adequately used, and a brute force strategy was applied to search for transcript-representing paths in the *de bruijn* graph, causing it to suffer seriously from false positive rates. Bridger successfully transplants the minimum path-cover model from the reference-based assembler Cufflinks to the de novo assembly and effectively avoids the exhaustive enumeration, making the false positives highly decreased. However, it does not make full use of the sequence depth information which should be useful in the development of assembling procedure as mentioned in the Trinity paper. Subsequently, a new assembler BinPacker was developed to fully use the sequence depth information by a bin-packing model without limiting the minimum number of paths. BinPacker performs better than others of same kind, but it has not integrated the paired-end information into the assembling procedure, leaving a big room to be improved.

In this paper, we introduce a new de novo assembler TransLiG developed by phasing paths and iteratively constructing weighted line graphs starting from splicing graphs. The idea of phasing paths in TransLiG was motivated from Scallop [13], a reference-based transcriptome assembler, which also adopted a similar strategy of phasing paths in a graph. Although Scallop and TransLiG shared the same idea of graph decomposition, they were differently using the sequence depth and paired-end information. Different from Scallop which decomposed graphs by iteratively constructing local bipartite graphs, TransLiG pursued the globally optimum solution by iteratively building weighted line graphs.

TransLiG was developed to integrate the sequence depth and pair-end information into the assembling procedure by phasing paths and iteratively constructing line graphs, making it substantially superior to all the salient tools of same kind, e.g., Trinity, Bridger, and BinPacker. When tested on both the artificial and real data, it reaches the precision 6% higher than BinPacker and Bridger, and nearly 15% higher than Trinity on artificial data, and 7%, 14%, and 21% higher than BinPacker, Bridger, and Trinity respectively, on the tested mouse data. Not only does TransLiG achieve the highest precision, but also it reaches the highest sensitivity on all the tested datasets. In addition, TransLiG stably keeps the best performance with different assessment parameters.

## Results and discussion

We compared TransLiG with five salient de novo assemblers: BinPacker (version 1.0), Bridger (version r2014-12-01), Trinity (version 13.02.25), IDBA-Tran (version 1.1.1), and SOAPdenovo-trans (version 1.0.3) on both artificial and real datasets. The parameter settings for each of them are described in the Additional file 1: Supplementary Notes.

## Assessment metrics and performance evaluation

Commonly used criteria were applied to the evaluation of all the salient de novo assembling algorithms in this experiment. Assembled transcripts by each assembler were aligned to the expressed transcripts in ground truth using BLAT [29] with 95% sequence identity as cutoff. An expressed transcript is called full-length reconstructed if it was covered by an assembled transcript with at least 95% sequence identity and no more than 5% insertions or deletions (indels), and this assembled transcript is called a true positive. To make more convincing comparisons, we also considered different sequence identity levels (90%, 85%, and 80%) to define full-length reconstructed reference transcripts and true positives. Accuracy is measured by sensitivity and precision, where sensitivity is defined as the number of full-length reconstructed transcripts in ground truth by an assembler, and precision is defined as the fraction of true positives out of all assembled transcripts.

## Evaluation on simulation data

We first tested TransLiG against the other assemblers on the simulation data which was generated by the tool Flux Simulator [30] using all the known human transcripts (approximately 83,000 sequences) from the UCSC hg19 gene annotation. The generated dataset contains approximately 55 million strand-specific RNA-seq paired-end reads of 76-bp length.

**Fig. 1:**
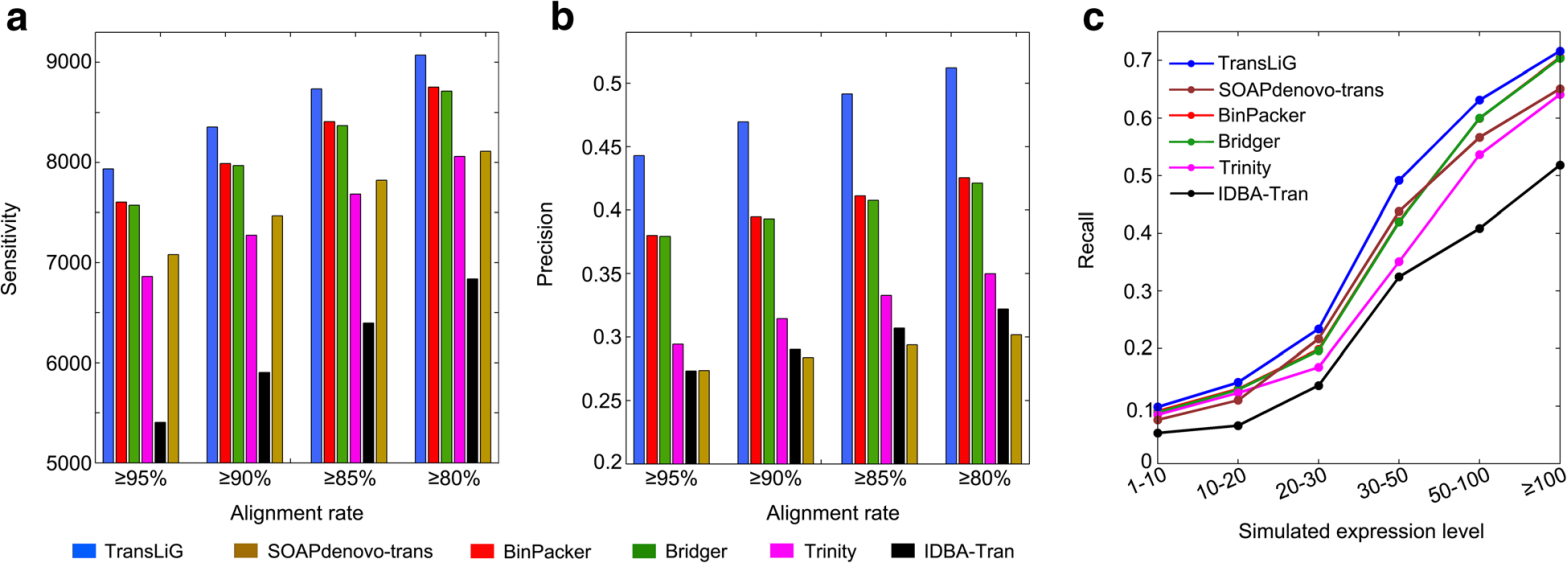
Comparison results on simulated data. **a** Comparison of sensitivity distributions of the six tools against different sequence identity levels. **b** Comparison of precision distributions of the six tools against different sequence identity levels. **c** Comparison of recall distributions of the six tools against transcript expression levels

Testing all the six assemblers on the simulation data, we found that TransLiG reconstructed 7935 full-length expressed transcripts, while BinPacker, Bridger, Trinity, IDBA-Tran, and SOAPdenovo-trans respectively recovered 7602, 7572, 6863, 5405, and 7080 full-length expressed transcripts. Therefore, TransLiG reaches the highest sensitivity followed by BinPacker and Bridger. The reason why BinPacker and Bridger are inferior to TransLiG while superior to the others is simply because they employed appropriately mathematical models in their assembly procedures, while they did not sufficiently use the paired-end and sequence depth information. Trinity was brutally enumerating all the paths over *de Bruijn* graphs, and thus generating a large amount of transcript candidates. However, the brute force strategy involves too many false positives, leading to a poor sensitivity. IDBA-Tran behaves the worst among all the compared tools. We can see from Fig. 1a that TransLiG recovered 4.38% more full-length expressed transcripts than the next best assembler BinPacker, and 15.62% more than Trinity. In addition, TransLiG consistently behaves the best in sensitivity under different sequence identity levels (95%, 90%, 85%, and 80%) as illustrated in Fig. 1a and Additional file 1: Table S1. Not only does TransLiG perform better in sensitivity than all the compared tools, but also it does in precision. It reaches the highest precision of 44.21% versus BinPacker of 38.01%, Bridger of 37.94%, Trinity of 29.43%, IDBA-Tran of 27.33%, and SOAPdenovo-trans of 27.37%, and keeps its superiority under different sequence identity levels (see Fig. 1b and Additional file 1: Table S1). SOAPdenovo-trans recovered more full-length expressed transcripts than Trinity, but its precision is the lowest among all the compared tools.

We also compared their abilities of reconstructing expressed full-length transcripts at different abundance levels, which was evaluated by *recall* defined as the fraction of full-length reconstructed expressed transcripts out of all expressed transcripts under different transcript abundance levels. We can see from Fig. 1c that IDBA-Tran shows the lowest recall under all the abundance levels, and the recalls of Trinity under low abundances (1–10 and 10–20) are similar with BinPacker and Bridger, a little higher than SOAPdenovo-trans. However, SOAPdenovo-trans demonstrates much higher recalls than Trinity under high abundances (20–100). TransLiG is consistently superior to all the compared tools in recall across all the expression levels.

We further compared the performance of the assemblers in identifying expressed genes. A gene is considered to be correctly identified if at least one of its transcripts was correctly assembled. We found that TransLiG correctly identified 6189 genes, while BinPacker, Bridger, Trinity, IDBA-Tran, and SOAPdenovo-trans identified 5984, 5979, 5247, 4865, and 5951, respectively. Therefore, TransLiG outperforms all the compared assemblers in terms of the number of expressed genes identified.

## Evaluation on real data

In this section, we tested the six assemblers on the following three real biological datasets, the human K562 cells, the human H1 cells, and the mouse dendritic cells datasets, containing 88 million, 41 million, and 53 million paired-end reads, respectively. They were collected from the NCBI Sequence Read Archive (SRA) database with accession codes SRX110318, SRX082572, and SRX062280, respectively. Different from simulated datasets, which provide us the explicit ground truth, it is impossible for us to know all the genuine transcripts encoded in the real datasets. Despite this, we collected all the currently known transcripts in the UCSC genome databases as references. The versions of human and mouse reference transcripts used in this study are GRCh37/hg19 and GRCm38/mm10, respectively.

**Fig. 2:**
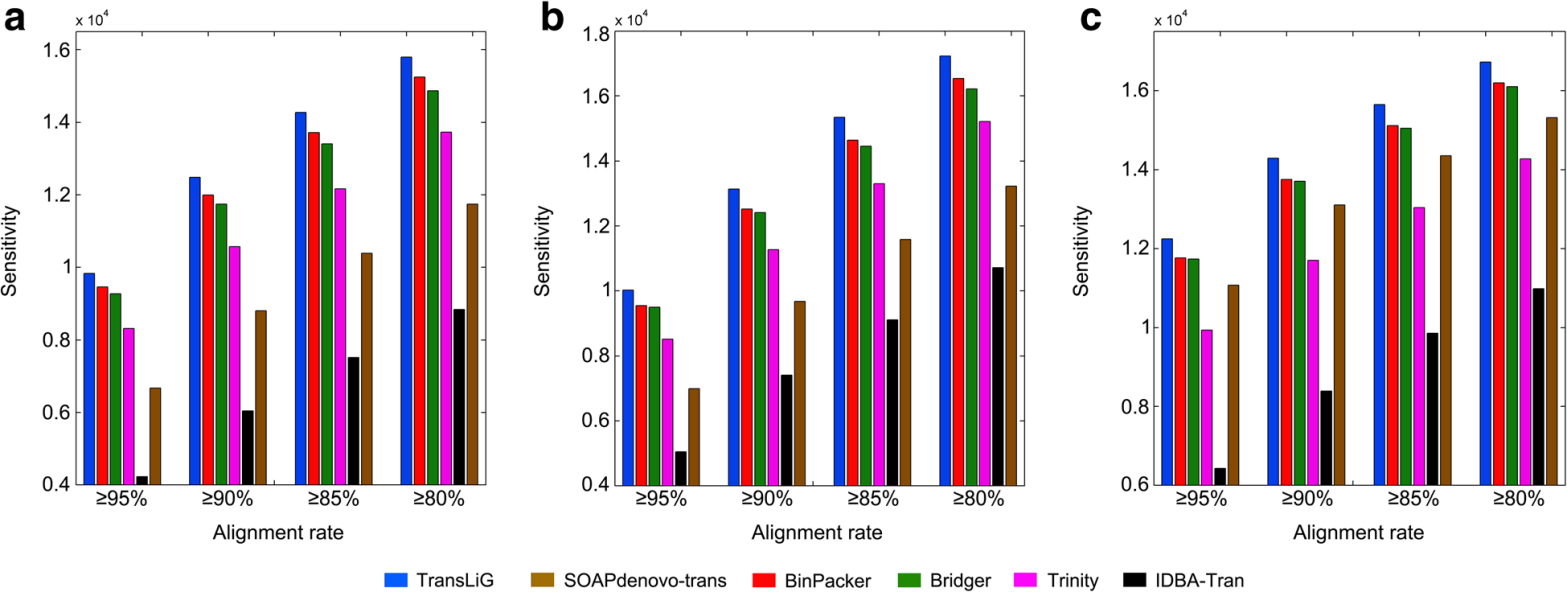
Comparison of sensitivity distributions of the six tools against the different sequence identity levels on the three real datasets: **a** human K562, **b** human H1, and **c** mouse dendritic

Running the six assembling tools on the human K562 cells, the human H1 cells and the mouse dendritic cells datasets, we found that TransLiG recovered 9826, 10,017, and 12,247 full-length reference transcripts respectively on the three real datasets, versus 9454, 9557, and 11,761 by the second best assembler BinPacker, and 8315, 8516, and 9937 by Trinity, i.e., TransLiG recovered 3.93%, 4.81%, and 4.13% more full-length reference transcripts than BinPacker, and 18.17%, 17.63%, and 23.25% more than Trinity. In addition, TransLiG consistently keeps the highest sensitivity under different sequence identity levels (see Fig. 2 and Additional file 1: Table S2-S4), clearly indicating its higher reliability and stability.

**Fig. 3:**
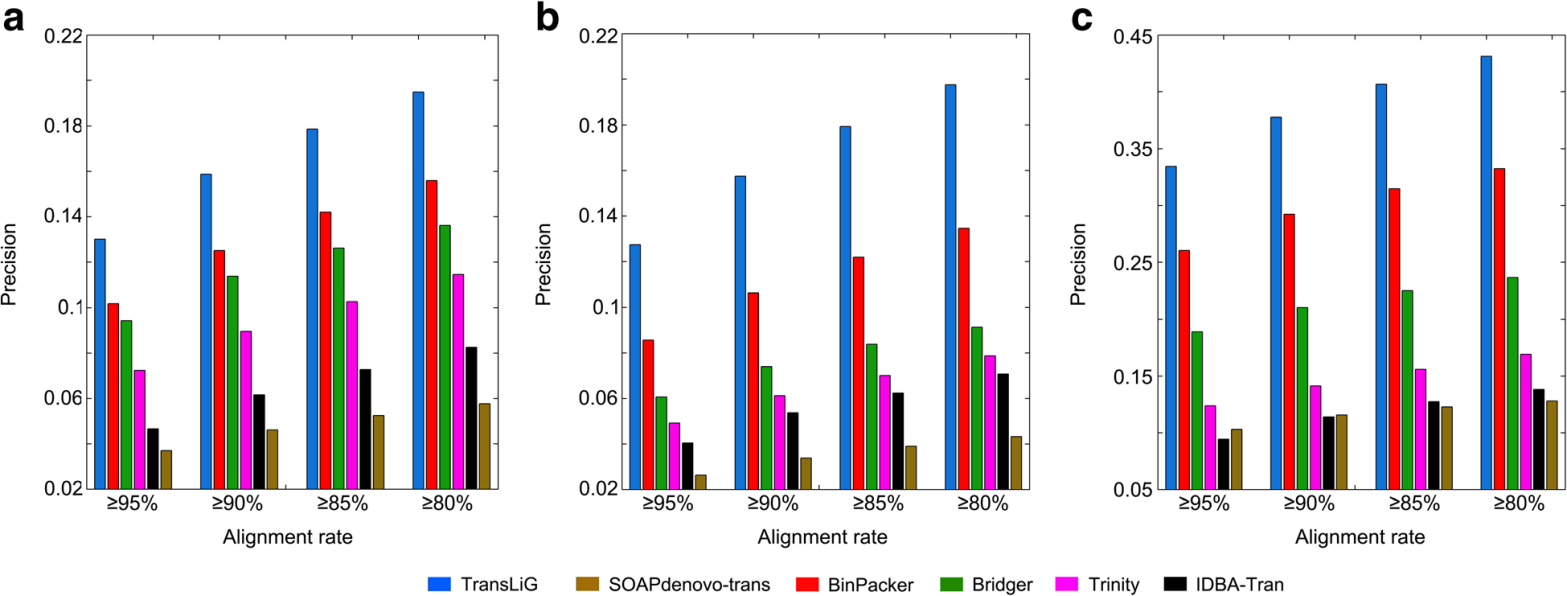
Comparison of precision distributions of the six tools against different sequence identity levels on the three real datasets: **a** human K562, **b** human H1, and **c** mouse dendritic

As for precision, TransLiG reaches 12.90%, 12.72%, and 33.26% on the human K562 cells, the human H1 cells and the mouse dendritic cells datasets, respectively, versus 10.18%, 8.57%, and 26.03% by the second best BinPacker, and 7.24%, 4.92%, and 12.37% by Trinity. Similar to the results on the simulation datasets, SOAPdenovo-trans shows the lowest precision among all the compared tools on the real datasets. By comparisons, we see that TransLiG has been significantly improved in precision compared to the others, especially on the mouse data, where the TransLiG achieves 7% more than the next best BinPacker, and 21% more than Trinity. In addition, the superiority of TransLiG was also clearly demonstrated by changing the sequence identity levels (see Fig. 3 and Additional file 1: Table S2-S4).

Similarly, we also compared the assemblers in terms of the numbers of the expressed genes identified on real datasets. We found that TransLiG correctly identified 5468, 5669, and 7425 genes on the human K562 cells, the human H1 cells, and the mouse dendritic cells datasets, respectively, versus 5359, 5485, and 7250 by BinPacker; 5330, 5509, and 7284 by Bridger; 4726, 5003, and 6262 by Trinity; 2823, 3387, and 4533 by IDBA-Tran; and 4179, 4403, and 7145 by SOAPdenovo-trans.

The evaluations on both artificial and real datasets have fully demonstrated that TransLiG consistently shows the best performance among all the salient tools of same kind no matter in terms of sensitivity, precision, or the number of identified genes.

## Evaluation of computing resource usage

**Fig. 4:**
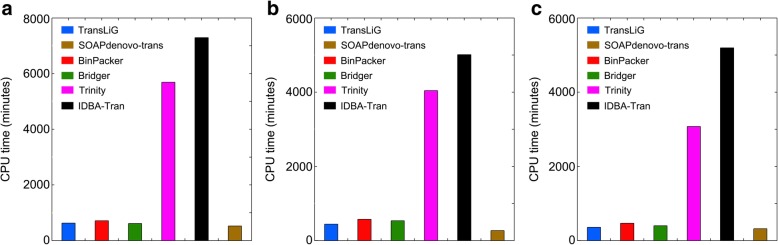
Comparison of CPU times for the six tools on the three datasets: **a** human K562, **b** human H1, and **c** mouse dendritic

**Fig. 5:**
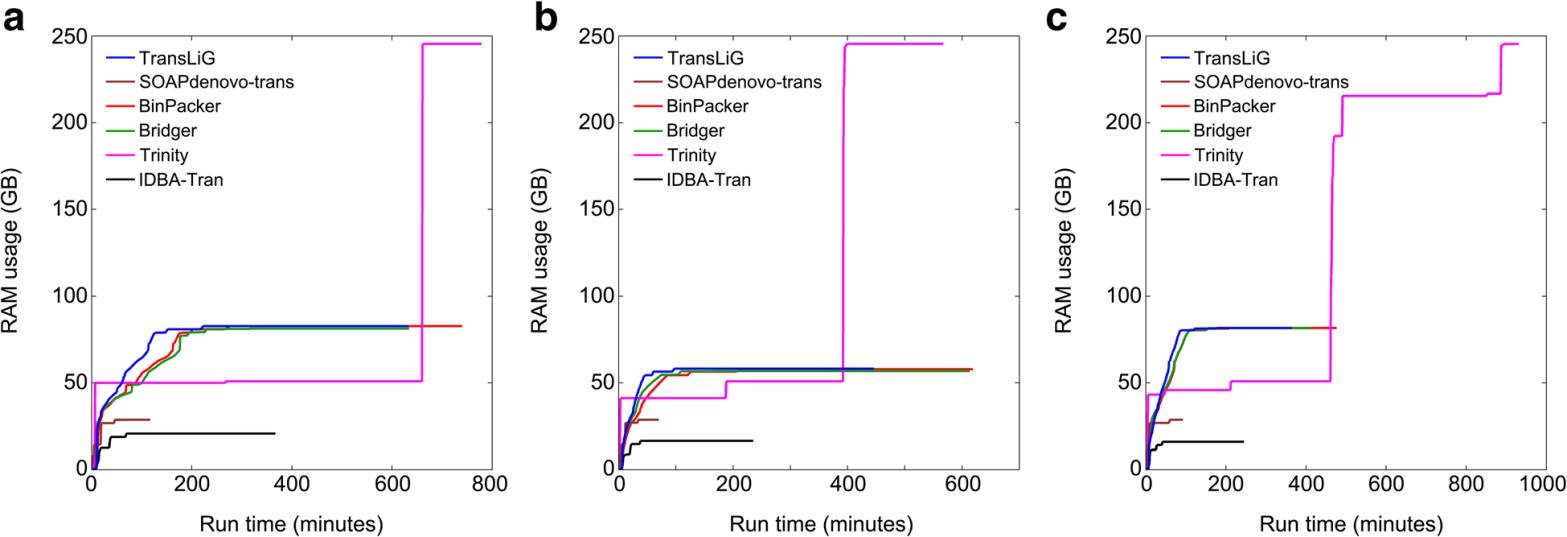
Comparison of RAM usages for the six tools on the three datasets: **a** human K562, **b** human H1, and **c** mouse dendritic

De novo assemblers generally consume large computing resources (e.g., CPU time and memory usage). We by Fig. 4 and Fig. 5 illustrate the CPU time and memory (RAM) usage by individual assemblers on the real datasets. We see from Fig. 4 that Trinity and IDBA-Tran consume much more CPU times than all the others on all the three datasets. SOAPdenovo-trans is the fastest one among all the compared tools, while TransLiG, BinPacker, and Bridger cost CPU times similar to SOAPdenovo-trans. As for the RAM usage, Fig. 5 shows that Trinity consumes much higher memory than all the others on all the three datasets, where TransLiG, BinPacker, and Bridger cost similar memory resources, but higher than IDBA-Tran and SOAPdenovo-trans. IDBA-Tran is the most parsimonious one followed by SOAPdenovo-trans in terms of RAM usage. Overall, TransLiG is not the most parsimonious, but it is quite acceptable for practical use.

## Conclusions

In this study, we presented a novel de novo assembler TransLiG for transcriptome assembly using short RNA-seq reads. Compared to the salient assemblers of same kind on both simulated and real datasets, TransLiG consistently performs the best in accuracy (including sensitivity and precision) and the number of correctly identified genes. The superiority may attribute to the following facts. Firstly, TransLiG constructs more accurate splicing graphs by reconnecting fragmented graphs via iterating different lengths of smaller k-mers. Secondly, TransLiG substantially integrates the sequence depth and paired-end information into the assembling procedure via enforcing each pair-supporting path being included in at least one assembled transcript. Thirdly, TransLiG accurately links the in-coming and out-going edges at each node via iteratively solving a series of quadratic programmings, which are optimizing the utilizations of the paired-end and sequencing depth information. Finally, TransLiG benefits from the iterations of weighted line graphs constructed by repeatedly phasing transcript-segment-representing paths. Notice that the final line graph *L*^*n*^(*G*) is empty, and the isolated nodes generated during the line graph iteration could be expanded into assembled transcripts.

To our best knowledge, TransLiG is the first de novo assembler which effectively integrates the paired-end and sequence depth information into the assembling procedure via phasing and contracting paths with the help of line graph iterations. The software has been developed to be user-friendly and expected to play a crucial role in new discoveries of transcriptome studies using RNA-seq data, especially in the research areas of complicated human diseases such as cancers, discoveries of new species, and so on.

## Methods

We designed the new de novo assembler TransLiG to retrieve all the transcript-representing paths in splicing graphs by phasing paths in the splicing graphs and iteratively constructing line graphs starting from the splicing graphs. For a graph *G* = (*V*, *E*), the line graph *L*(*G*) of *G* is the graph with nodes representing edges of *G*, and edges representing incident relationship between edges in *G*, i.e., two nodes *u, v* of *L(G)*, that are edges of *G*, are connected by an edge in *L(G)* if and only if they share a node in *G*. Obviously, the line graph of a directed acyclic graph (DAG) remains a DAG. Therefore, the line graph of a splicing graph must be a DAG. By *L*^*n*^(*G*), we define the line graph of *G* of order *n*, i.e., *L*^*n*^(*G*) = *L*(*L*^*n*-1^(*G*)), where *L*^0^(*G*) = *G*. It then turns out that *L*^*n*^(*G*) is an empty graph, i.e., a graph with all nodes being isolated for a DAG graph *G* of *n* nodes. Obviously, each isolated node generated during the line graph iteration can be expanded into a transcript-representing path *P* in *G*.

**Fig. 6:**
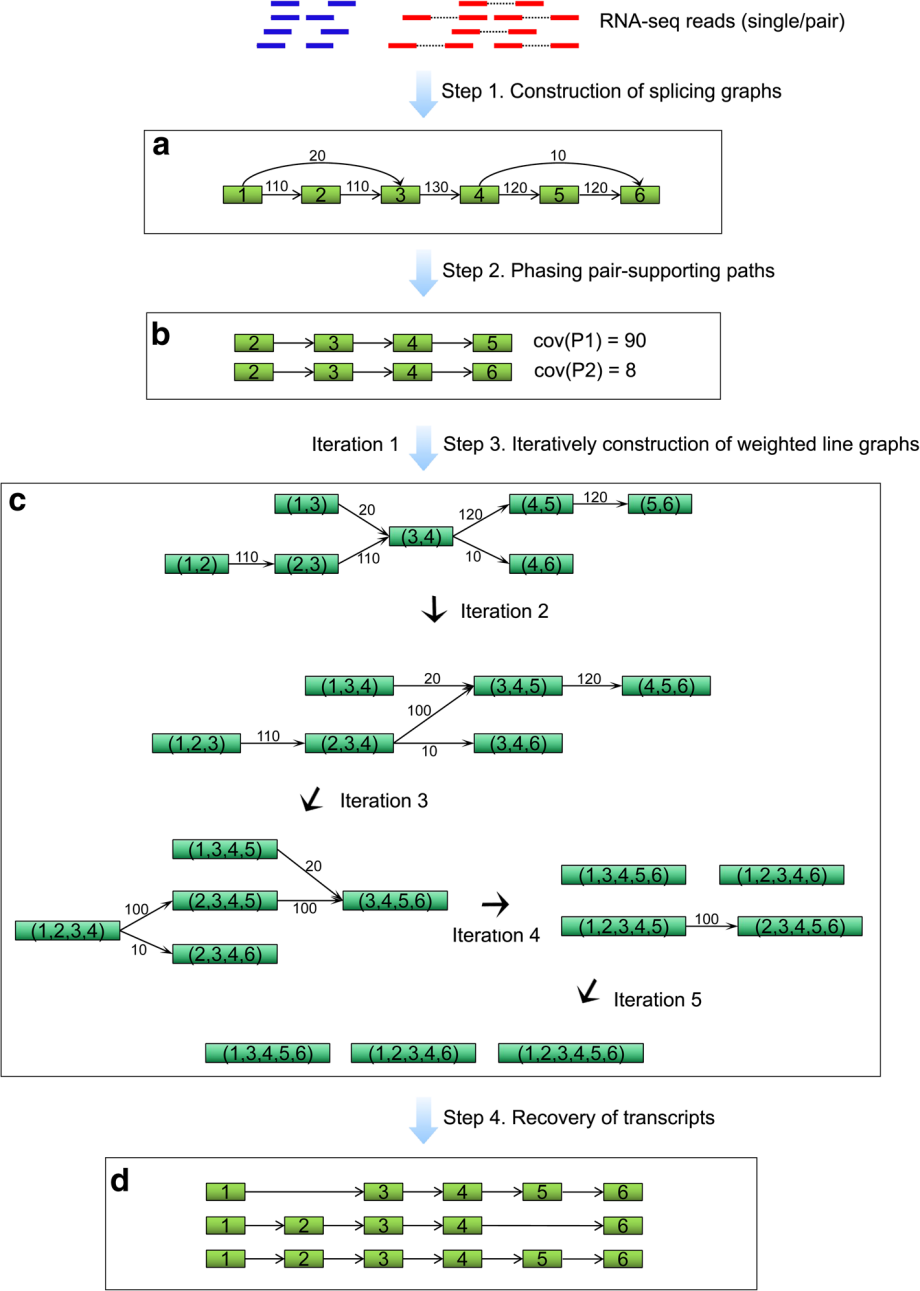
Flowchart of TransLiG. **a** TransLiG takes as input the RNA-seq reads (single or pair) to construct splicing graphs by first using the graph-building framework of BinPacker to build initial splicing graphs, and then modifying initial splicing graphs by merging the isolated pieces in a specific way. **b** TransLiG phases pair-supporting paths from the splicing graphs to ensure that each pair-supporting path is covered by an assembled transcript. **c** TransLiG assembles transcripts by iteratively constructing weighted line graphs until empty. **d** The transcript-representing paths are obtained by expanding all the isolated nodes generated during the line graph iteration

The basic idea behind TransLiG is to globally optimize the accuracy of retrieving all the full-length transcripts encoded in a splicing graph by phasing paths and iteratively constructing the next line graph *L*^*i +* 1^(*G*) weighted by solving series of quadratic programming problems defined on the current (line) graph *L*^*i*^(*G*). After removing all the zero-weighted edges from the constructed line graph, the remaining graph ideally consists of the line graph edges of individual transcript-representing paths in the current graph *L*^*i*^(*G*). Continuing the iteration until *L*^*n*^(*G*) becomes empty. Hopefully, each isolated node generated during the line graph iteration will be expanded into a transcript-representing path, which exactly corresponds to an expressed transcript. The flowchart of the TransLiG is roughly outlined in Fig. 6 and followed by the pseudo-codes of TransLiG.

## De novo assembling algorithm TransLiG

### Step 1: Construction of splicing graphs

We first construct initial splicing graphs based on the graph-building framework of BinPacker [23], and then, we designed a novel technique to effectively modify the initial splicing graphs by merging the isolated pieces (see Additional file 1: Methods 2.1 for details).

### Step 2: Phasing pair-supporting paths

Ideally, each expressed transcript *T* corresponds to a unique path *P*_*T*_ in the splicing graph, and so a segment in the transcript *T* corresponds to a sub-path of *P*_*T*_, which is called a transcript-segment-representing path later on. Two paired-end reads *r*_1_ and *r*_2_ are supposed to come from a single transcript, corresponding to a segment in the transcript, and so a transcript-segment-representing path in the splicing graph. The transcript-segment-representing path in this paper is called a pair-supporting path. TransLiG retrieves a set, denoted by *P*_*G*_, of all the pair-supporting paths for each splicing graph *G*.

In detail, for each single-end read *r*, if it spans a sub-path *P* = *n*_*i*1_ → *n*_*i*2_ → … → *n*_*ip*_ of graph *G* and *p* ≥ 3, then we add a pair-supporting path *P* = *n*_*i*1_ → *n*_*i*2_ → … → *n*_*ip*_ to *P*_*G*_. For the case of paired-end reads *r*_1_ and *r*_2_, if *r*_1_ spans a sub-path *P*_1_ = *n*_*i*1_ → *n*_*i*2_ → … → *n*_*ip*_, and *r*_2_ spans a sub-path *P*_2_ = *n*_*j*1_ → *n*_*j*2_ → … → *n*_*jq*_, and there exists a unique path *P*_*in*_ = *n*_*ip*_ → *n*_*m*1_ → *n*_*m*2_ → … → *n*_*mk*_ → *n*_j1_ between *n*_*ip*_ and *n*_*j*1_ in *G*, and *p* + *k* + *q* ≥ 3, we then add a pair-supporting path *P* = *P*_1_ → *P*_*in*_ → *P*_2_ to *P*_*G*_. Different reads (single-end or paired-end) may generate the same pair-supporting path. So, each pair-supporting path *P* is assigned a weight *cov*(*P*) as the number of reads that could generate the path *P*.

### Step 3: Iteratively construction of weighted line graphs

Starting from a splicing graph, let *G* be the current weighted (line) graph. TransLiG weights the line graph *L*(*G*) of *G* via solving a quadratic program at each node of *G*. Assume that node *v* in *G* has *n* in-coming edges and *m* out-going edges. In theory, there are *m* × *n* feasible connections between the *n* in-coming edges and *m* out-going edges. We expect to find the correct connections that the to-be-assembled transcripts pass through. To achieve this goal, we designed the following programming, in which ${\left({s}_j-\sum \limits_{j=1,\dots, m}{w}_{ij}{x}_{ij}\right)}^2$ measures the deviation between the weight of the in-coming edge *e*_*i*_ and the sum of the weights of all the transcript-representing paths passing through *e*_*i*_, and similarly for ${\left({\mathrm{c}}_j-\sum \limits_{i=1,\dots, n}{w}_{ij}{x}_{ij}\right)}^2$, where *s*_*i*_ is the weight of the in-coming edge *e*_*i*_ at *v*, *c*_*j*_ the weight of the out-going edge *e*_*j*_ at *v*; *x*_*ij*_ represents a binary variable with *x*_*ij*_ = 1 if there is at least one transcript-representing path passing through *e*_*i*_ and *e*_*j*_, and 0 otherwise, and *w*_*ij*_ represents the coverage value of all the transcript-representing paths passing through *e*_*i*_ and *e*_*j*_. We then minimize the deviations for all the in-coming and out-going edges to find the correct connections between the in-coming and out-going edges. Therefore, the correct way of all the transcript-representing paths passing through the node *v* could be determined by solving the following quadratic program.

$$ {\displaystyle \begin{array}{c}\min \kern0.5em z=\sum \limits_{i=1,\dots, n}{\left({s}_i-\sum \limits_{j=1,\dots, m}{w}_{ij}{x}_{ij}\right)}^2+\sum \limits_{j=1,\dots, m}{\left({c}_j-\sum \limits_{i=1,\dots, n}{w}_{ij}{x}_{ij}\right)}^2\\ {}s.t.\kern0.5em \left\{\begin{array}{c}\begin{array}{ccccccc}{x}_{ij}=1,& if& \left({e}_i,{e}_j\right)\subset P,P\in {P}_{\mathrm{G}}& & & & \end{array}\\ {}\begin{array}{cc}{w}_{ij}\ge \sum \limits_{P\in {P}_G,\left({e}_i,{e}_j\right)\subset P}\operatorname{cov}(P),& \begin{array}{cc}i=1,\dots, n,& j=1,\dots, m\end{array}\end{array}\\ {}\begin{array}{cc}\sum \limits_{i=1,\dots, n}{x}_{ij}\ge 1,& j=1,\dots, m\end{array}\\ {}\begin{array}{cc}\sum \limits_{j=1,\dots, m}{x}_{ij}\ge 1,& i=1,\dots, n\end{array}\\ {}{w}_{ij}\ge 0\\ {}\begin{array}{c}{x}_{ij}=\left\{0,1\right\}\\ {}\sum \limits_{\begin{array}{c}i=1,\dots, n\\ {}j=1,\dots, m\end{array}}{x}_{ij}=M\end{array}\end{array}\right.\end{array}} $$

where *P*_*G*_ is the set of pair-supporting paths, and *cov*(*P*) is the coverage of pair-supporting path *P*; *M* represents the expected number of transcript-representing paths passing through the node *v*, and clearly *max*{*m*, *n*} ≤ *M* ≤ *m* × *n* (see Additional file 1: Methods 2.2 in details for the determination of *M*). Clearly, this is a mixed integer quadratic programming, an NP-hard problem. However, it is computationally acceptable in our assembly procedure due to the specific properties of the constructed splicing graphs (see Additional file 1: Methods 2.3 in details for the solution of the quadratic programming).

Let {*x*_*ij*_, *w*_*ij*_} be the optimum solution of the quadratic program. Then the line graph *L*(*G*) of the current (line) graph *G* is weighted by assigning *w*_*ij*_ to (*e*_*i*_, *e*_*j*_) if *x*_*ij*_ = 1, and 0 otherwise. Reset *G* to be the graph *L*(*G*) by removing all the zero-weighted edges, and modifying the pair-supporting paths in *P*_*G*_ accordingly. Then TransLiG repeats Step 3 until *L*(*G*) is empty.

### Step 4: Recovery of transcripts

TransLiG recovers all the transcripts by expanding all the isolated nodes generated during the line graph iteration, i.e., by tracking back to recover all the transcript-representing paths in the original splicing graphs.

## Additional file

Additional file 1: This file contains the parameter setups of the compared assemblers, the supplementary methods, figures and tables. (PDF 368 kb)

## Abbreviations

NCBI: National Center for Biotechnology Information
RNA-seq: RNA sequencing
SRA: Sequence read archive
