# Supplementary material for: TransLiG: a de novo transcriptome assembler that uses line graph iteration
Source: Genome Biol. 2019 Apr 23;20:81. doi: 10.1186/s13059-019-1690-7 (PMC6480747; doi:10.1186/s13059-019-1690-7)
Supplement: Supplementary file 1 — This file contains the parameter setups of the compared assemblers, the supplementary methods, figures and tables. (PDF 368 kb) [file 13059_2019_1690_MOESM1_ESM.pdf]

Supplementary Material for the Paper

TransLiG: a de novo transcriptome assembler that uses line graph iteration

Juntao Liu[†], Ting Yu[†], Zengchao Mu, Guojun Li[*]

School of Mathematics, Shandong University, Jinan 250100, China

1. Supplementary Notes

1.1 Parameter setup of the compared assemblers

Parameters are set up as follows for each algorithm: **BinPacker** (version 1.0): k=31 and “-- SS_lib_type FR” for simulation data and “-- SS_lib_type RF” real data; **Bridger** (version r2014-12-01): k=31 and “-- SS_lib_type FR” for simulation data and “-- SS_lib_type RF” real data; **Trinity** (version 13.02.25): “--CPU 10 --JM 50G --SS_lib_type FR” for simulation data and “--CPU 10 --JM 50G --SS_lib_type RF” for real data; **IDBA-Tran** (version 1.1.1): “-mink 21 -maxk 37 -step 4” for all the tested data; **SOAPdenovo-trans** (version 1.0.3) was run using default parameters. All the assemblers were performed on a server with 768GB of RAM.

2. Supplementary Methods

2.1. Modifying initial splicing graphs

Note that the splicing graphs constructed by BinPacker cannot be obtained by a contracted *de Bruijn* graph. One example showing their differences can be found in Additional file 1: Figure S1. In addition, the *de Bruijn* graph usually suffers a problem that the first graph built from the hash table is very huge because many genes are mixed together by sharing their *k*-mers. However, the splicing graphs constructed here could keep their size smaller by using paired-read information to check if a new branch should be added, which makes finding transcripts from the graph much easier.

The graph-building framework of BinPacker is based on an extension method using overlaps between length-fixed k-mer (e.g. k = 31). It has been observed that longer k-mer will result in a more contiguous assembly for highly expressed transcripts, meanwhile, a large amount of fragmented sequences for lowly expressed ones. However, lowly expressed

transcripts will be better assembled if shorter k-mer are used, unfortunately, in this case the graphs of highly expressed ones will be quite complicated with many false connections. Based on the observations mentioned above, we first use a relatively longer k-mer to construct initial splicing graphs, and then modify the initial splicing graphs by connecting the fragmented sequences using shorter k-mers. The modifying process of the initial splicing graphs is elaborated below.

Step 1. Collecting connections between splicing graphs using smaller kmers. A node without in-coming or out-going edges is called a leaf of the splicing graph. Due to the existence of sequencing errors, the terminal of a leaf node may contain a short sequence erroneously extended, and therefore the last k bps (smaller than K_{raw}, the length of k-mer used for building initial splicing graphs) of node N_1 and the first k bps of node N_2 may be different though they should be connected together. In order to handle this, we choose the first L bps (default as K_{raw}) of each leaf node without in-coming edges, and last L bps of each leaf node without out-going edges, denoted by K_f and $K_{l,}$, respectively. Then we build candidate connections between leaf nodes by iteratively searching for overlapping k-mers of length from K_{raw} - 2 to k_{min} in the K_f and K_l sequences. In detail, if there are m ($m \geq 1$) k-mers of lengths $k_1, k_2, \ldots, k_m$ ($k_{min} \leq k_i \leq K_{raw} - 2$) simultaneously occurring in the K_l sequence of leaf node n_i and the K_f sequence of leaf node n_j, then we record a connection (n_i, n_j) with its maximum length of the m k-mers.

Step 2. Filteration of erroneous connections between leaf nodes. Since all the collected connections between leaf nodes are supported by a smaller k-mer overlap, it is easily to introduce false positives. In order to ensure correctness, we keep only one connection with the highest reliability for each leaf node. In detail, we first choose the leaf node N_1 with the largest node coverage and connect it to its best neighbor N_2, defined as owning the maximum overlapping k-mer length (first priority) and largest node coverage (second priority). The coverage of the new edge is defined as the smaller node coverage of the two leaf nodes. Then we remove all the other connections related to N_1 and N_2, and choose another leaf node with the largest node coverage and repeat the above procedure till no connections left. After that, two or more fragmented splicing graphs might be connected into a single graph.

2.2. Determination of optimum M in the quadratic programming

In order to obtain the optimum M, we started M from $max\{m, n\}$ and iteratively increase its value and stop at the optimum one. Given M, the quadratic programming could be solved. After obtaining the optimum solution $\{x_{ij}, w_{ij}\}$ and the minimum objective value $z = z_{min}$, we increased M by 1 and resolve the quadratic programming and obtain the optimum solution $\{x_{ij}', w_{ij}'\}$ and minimum $z = z_{min}'$. If $z_{min}' \leq \varepsilon \cdot z_{min}$ (the default value of ε is set to be 0.5), which means that M+1 shows significantly smaller objective value than M does, then $\{x_{ij}, w_{ij}\}$ was updated to $\{x_{ij}', w_{ij}'\}$, and we again increase M by 1 and resolve the quadratic programming. Keep increasing M until $z_{min}' > \varepsilon \cdot z_{min}$, and finally we obtain the optimum M and the global optimum solution $\{x_{ij}, w_{ij}\}$.

2.3. Solution of the quadratic programming

In order to effectively solve this mixed integer quadratic programming in the main text, we attempted to convert it into a quadratic programming without integer constraints as follows. Firstly, enumerate all the combinations of choosing M sub-paths from the $n \cdot m$ ones. And then delete those combinations which are incompatible with the constraints, e.g. the combinations without considering all the pair-supporting paths, or without covering all the in-coming and out-going edges. Then each of the remaining combinations corresponds to a specific value of $\{x_{ij}\}$, and so a quadratic programming without integer constraints. After solving each programming, choose the optimum $\{x_{ij}\}$ with the minimum objective value. Theoretically, this step is very time-consuming. However, a large fraction of incompatible combinations have been deleted first, and moreover, quite a amount of edges with low reliability are also removed during the graph construction, making m, $n \leq 5$ and $n \cdot m \leq 20$ in most cases. So, the scale of the quadratic programming is not very large, which could be solved ultra fast.

3. Supplementary Figures

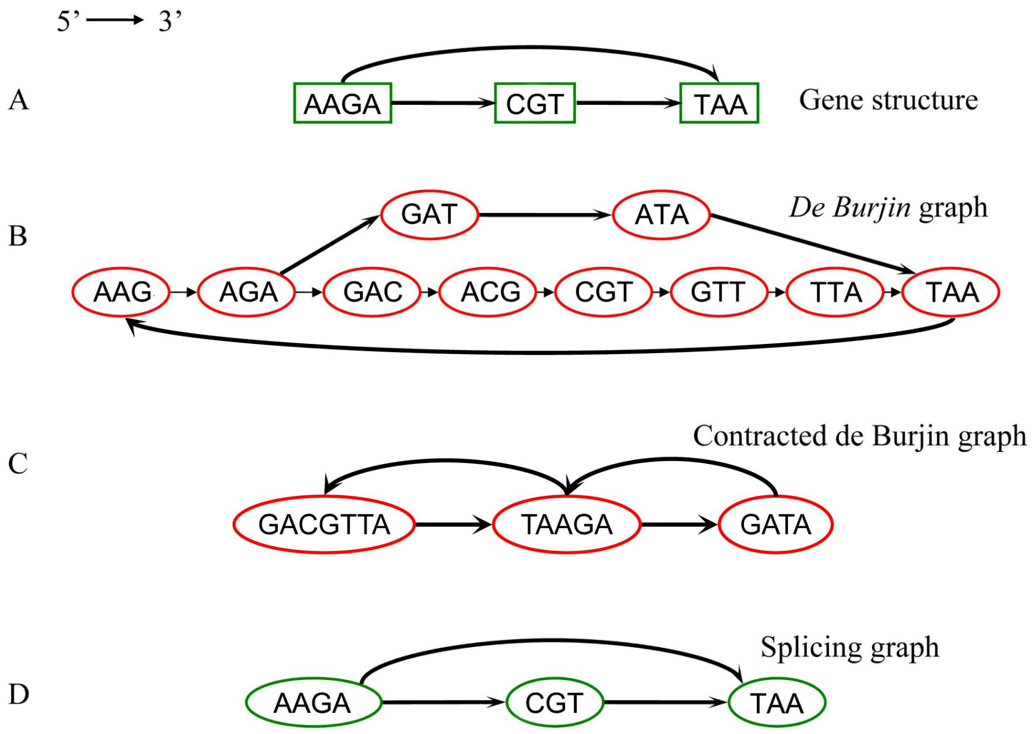

Figure S1. One example shows that the splicing graph is different from contracted *de Bruijn* graph. (A) gene structure with two isoforms, (B) *de Bruijn* graph, (C) contracted *de Bruijn* graph, (D) splicing graph.

4. Supplementary Tables

Table S1. Assembly accuracy of the de novo assemblers on simulated data.

**Simulation**	**Alignment rate**			
**TransLiG**	**0.95**	**0.9**	**0.85**	**0.8**
**Candidates**	17226	17226	17226	17226
**Full-length recovered**	7935	8355	8736	9072
**Ture positives**	7616	8075	8452	8805
**Precision**	44.21%	46.88%	49.07%	51.11%
**BinPacker**	**0.95**	**0.9**	**0.85**	**0.8**
**Candidates**	17862	17862	17862	17862
**Full-length recovered**	7602	7990	8409	8754
**Ture positives**	6789	7053	7342	7598
**Precision**	38.01%	39.49%	41.10%	42.54%
**Bridger**	**0.95**	**0.9**	**0.85**	**0.8**
**Candidates**	17382	17382	17382	17382
**Full-length recovered**	7572	7969	8369	8714
**Ture positives**	6594	6833	7084	7322

**Precision**	37.94%	39.31%	40.75%	42.12%
**Trinity**	**0.95**	**0.9**	**0.85**	**0.8**
**Candidates**	24784	24784	24784	24784
**Full-length recovered**	6863	7275	7684	8060
**Ture positives**	7295	7792	8248	8673
**Precision**	29.43%	31.44%	33.28%	34.99%
**IDBA-Tran**	**0.95**	**0.9**	**0.85**	**0.8**
**Candidates**	17159	17159	17159	17159
**Full-length recovered**	5405	5905	6395	6837
**Ture positives**	4690	4980	5268	5526
**Precision**	27.33%	29.02%	30.70%	32.20%
**SOAPdenovo-trans**	**0.95**	**0.9**	**0.85**	**0.8**
**Candidates**	22300	22300	22300	22300
**Full-length recovered**	7080	7470	7822	8111
**Ture positives**	6103	6330	6551	6729
**Precision**	27.37%	28.39%	29.38%	30.17%

Table S2. Assembly accuracy of the *de novo* assemblers on human K562 data.

**Human K562**	**Alignment rate**			
**TransLiG**	**0.95**	**0.9**	**0.85**	**0.8**
**Candidates**	65065	65065	65065	65065
**Full-length recovered**	9826	12482	14279	15794
**Ture positives**	8395	10242	11532	12580
**Precision**	12.90%	15.74%	17.72%	19.33%
**BinPacker**	**0.95**	**0.9**	**0.85**	**0.8**
**Candidates**	76294	76294	76294	76294
**Full-length recovered**	9454	11994	13721	15243
**Ture positives**	7770	9543	10834	11905
**Precision**	10.18%	12.51%	14.20%	15.60%
**Bridger**	**0.95**	**0.9**	**0.85**	**0.8**
**Candidates**	70130	70130	70130	70130
**Full-length recovered**	9265	11741	13406	14862
**Ture positives**	6612	7974	8850	9549
**Precision**	9.43%	11.37%	12.62%	13.62%
**Trinity**	**0.95**	**0.9**	**0.85**	**0.8**
**Candidates**	104744	104744	104744	104744
**Full-length recovered**	8315	10568	12165	13730
**Ture positives**	7587	9381	10760	11993

**Precision**	7.24%	8.96%	10.27%	11.45%
**IDBA-Tran**	**0.95**	**0.9**	**0.85**	**0.8**
**Candidates**	52317	52317	52317	52317
**Full-length recovered**	4224	6038	7518	8843
**Ture positives**	2437	3223	3811	4321
**Precision**	4.66%	6.16%	7.28%	8.26%
**SOAPdenovo-trans**	**0.95**	**0.9**	**0.85**	**0.8**
**Candidates**	112150	112150	112150	112150
**Full-length recovered**	6669	8806	10383	11741
**Ture positives**	4154	5169	5893	6473
**Precision**	3.70%	4.61%	5.25%	5.77%

Table S3. Assembly accuracy of the *de novo* assemblers on human H1 data.

**Human H1**	**Alignment rate**			
**TransLiG**	**0.95**	**0.9**	**0.85**	**0.8**
**Candidates**	64635	64635	64635	64635
**Full-length recovered**	10017	13140	15351	17232
**Ture positives**	8224	10166	11578	12745
**Precision**	12.72%	15.73%	17.91%	19.72%
**BinPacker**	**0.95**	**0.9**	**0.85**	**0.8**
**Candidates**	85383	85383	85383	85383
**Full-length recovered**	9557	12523	14643	16538
**Ture positives**	7321	9069	10410	11495
**Precision**	8.57%	10.62%	12.19%	13.46%
**Bridger**	**0.95**	**0.9**	**0.85**	**0.8**
**Candidates**	107588	107588	107588	107588
**Full-length recovered**	9507	12415	14462	16214
**Ture positives**	6516	7961	9018	9824
**Precision**	6.06%	7.40%	8.38%	9.13%
**Trinity**	**0.95**	**0.9**	**0.85**	**0.8**
**Candidates**	161682	161682	161682	161682
**Full-length recovered**	8516	11264	13302	15220
**Ture positives**	7949	9890	11339	12731
**Precision**	4.92%	6.12%	7.01%	7.87%
**IDBA-Tran**	**0.95**	**0.9**	**0.85**	**0.8**
**Candidates**	74949	74949	74949	74949
**Full-length recovered**	5044	7406	9113	10712
**Ture positives**	3029	4022	4678	5298
**Precision**	4.04%	5.37%	6.24%	7.07%

SOAPdenovo-trans	0.95	0.9	0.85	0.8
**Candidates**	157208	157208	157208	157208
**Full-length recovered**	6991	9684	11582	13226
**Ture positives**	4137	5318	6124	6793
**Precision**	2.63%	3.38%	3.90%	4.32%

Table S4. Assembly accuracy of the *de novo* assemblers on mouse dendritic data.

Mouse dendritic	Alignment rate			
**TransLiG**	**0.95**	**0.9**	**0.85**	**0.8**
**Candidates**	32859	32859	32859	32859
**Full-length recovered**	12247	14293	15656	16726
**Ture positives**	10929	12353	13298	14108
**Precision**	33.26%	37.59%	40.47%	42.93%
**BinPacker**	**0.95**	**0.9**	**0.85**	**0.8**
**Candidates**	37219	37219	37219	37219
**Full-length recovered**	11761	13752	15123	16195
**Ture positives**	9687	10878	11720	12380
**Precision**	26.03%	29.23%	31.49%	33.26%
**Bridger**	**0.95**	**0.9**	**0.85**	**0.8**
**Candidates**	47541	47541	47541	47541
**Full-length recovered**	11734	13710	15053	16104
**Ture positives**	8989	9998	10696	11247
**Precision**	18.91%	21.03%	22.50%	23.66%
**Trinity**	**0.95**	**0.9**	**0.85**	**0.8**
**Candidates**	77887	77887	77887	77887
**Full-length recovered**	9937	11701	13040	14280
**Ture positives**	9636	11006	12139	13167
**Precision**	12.37%	14.13%	15.59%	16.91%
**IDBA-Tran**	**0.95**	**0.9**	**0.85**	**0.8**
**Candidates**	44920	44920	44920	44920
**Full-length recovered**	6426	8389	9858	10981
**Ture positives**	4239	5121	5728	6214
**Precision**	9.44%	11.40%	12.75%	13.83%
**SOAPdenovo-trans**	**0.95**	**0.9**	**0.85**	**0.8**
**Candidates**	69012	69012	69012	69012
**Full-length recovered**	11070	13104	14357	15326
**Ture positives**	7110	7988	8472	8835
**Precision**	10.30%	11.57%	12.28%	12.80%

1. Grabherr MG, Haas BJ, Yassour M, Levin JZ, Thompson DA, et al. (2011) Full-length transcriptome assembly from RNA-Seq data without a reference genome. Nat Biotechnol 29: 644-652.
2. CE S (1951) Prediction and entropy of printed English. Bell system technical journal: 50-64.
